# Advancing bone health evaluation in Hermann’s tortoises (*Testudo hermanni*) using spectral detector computed tomography

**DOI:** 10.3389/fvets.2026.1772226

**Published:** 2026-03-16

**Authors:** A. Hörmann, J. Hetterich, T. Neubert, G. Campbell, K. Merhof, M. Pees

**Affiliations:** 1 Department of Small Animal Medicine and Surgery, University of Veterinary Medicine Hannover, Hanover, Germany; 2 Department of Small Mammal, Reptile and Avian Medicine and Surgery, University of Veterinary Medicine Hannover, Hanover, Germany; 3 Faculty of Forest Sciences and Forest Ecology, Georg-August University of Göttingen, Göttingen, Germany; 4 Clinical Science, Philips, Hamburg, Germany

**Keywords:** BMD, bone mineral density, Hermann’s tortoise, MBD, metabolicbone disease, SDCT, spectral computed tomography, *Testudo hermanni*

## Abstract

**Introduction:**

Metabolic bone disease (MBD) is a common disorder in captive chelonians. Early diagnosis is challenging due to non-specific clinical signs, normal blood values in early stages, and limited sensitivity of radiographs. Spectral detector computed tomography (SDCT) offers quantitative, phantomless assessment of bone mineral density (BMD) and may improve diagnostic accuracy. This study evaluated SDCT’s feasibility for measuring BMD in Hermann’s tortoises (*Testudo hermanni*) and its ability to distinguish healthy from MBD-affected individuals. We also examined whether CT-derived shell morphometric ratios correlate with shell softening severity.

**Materials and methods:**

Twenty-three tortoises were divided into healthy and MBD-affected groups, with shell softness graded subjectively. SDCT scans were acquired without sedation, and BMD maps were generated using a phantom-based linear calibration model. Regions of interest were placed at the gular scute, scapula, and ilium. BMD distributions, mean values, and shell morphometric ratios were compared between groups.

**Results:**

106 measurement sites were analyzed. SDCT detected significant BMD declines from healthy to severely MBD-affected tortoises (*p* ≤ 0.05) at all sites, most pronounced at the ilium. Diseased animals showed right-skewed BMD histograms with low-attenuation pixel accumulation. Shell height-to-width ratios did not differ significantly. BMD generally reflected clinical status, though mild cases overlapped with healthy values.

**Conclusion:**

SDCT is a sensitive, non-invasive method for detecting and quantifying demineralisation in Hermann’s tortoises. It distinguishes healthy from MBD-affected individuals, enables objective observer-independent assessment, and complements clinical examination. SDCT shows strong potential for early diagnosis and monitoring of treatment in chelonian MBD. Larger, age-stratified cohorts are needed to confirm these findings.

## 1 Introduction

In recent years, there has been a notable increase in the popularity of reptiles as domestic animals across Europe (1, 2). The rise in the pet reptile population has been accompanied by an increase in husbandry-related health problems (3–5). Metabolic bone disease (MBD) is one of the most frequently observed disorders in captive reptiles (6, 7). Nutritional and environmental factors associated with improper husbandry have been demonstrated to impair bone mineralization, resulting in weakened skeletal structures and deformities. Other terms which may be used for this disease syndrome include fibrous osteodystrophy, osteomalacia, secondary nutritional hyperparathyroidism, osteoporosis, and rickets (8, 9).

The etiology of MBD is multifactorial, involving disturbances in calcium (Ca), phosphorus (P), and vitamin D3 metabolism (6). These disturbances are commonly caused by inadequate diets, including a low Ca content, a negative Ca-to-P ratio, or vitamin D3 deficiency (6). Insufficient environmental management, including improper light cycles, lack of ultraviolet B exposure, and suboptimal temperature and humidity levels, can contribute to or worsen the development of MBD (5).

A dietary Ca-to-P ratio of 1.5 to 2.0:1 is recommended for reptiles, with higher Ca needs for juveniles and reproductively active females (10). Hypocalcaemia can have other causes, such as renal disease, which can also cause secondary hyperparathyroidism and must be distinguished from MBD because of similar symptoms. MBD can be avoided through proper diet and environment, making educating tortoise caretakers essential (5).

Diagnosing MBD requires a thorough clinical history and physical examination, supported by diagnostic tests such as hematology and plasma biochemistry, where decreased Ca levels and increased P levels are often seen (9). However, Ca and P levels may remain normal in early-stage MBD and even in symptomatic tortoises. An imbalance in the Ca: P ratio causes secondary hyperparathyroidism, which promotes bone Ca resorption. Without replenishing Ca, inorganic bone tissue decreases, leading to the formation of fibrous tissue (11, 12).

Clinical signs of MBD, such as depression, lethargy or anorexia, can be subtle and nonspecific and may not be recognized by caretakers, especially in early stages. However, MBD can also lead to more obvious skeletal manifestations, such as softening of the carapace or plastron, shell deformities, and growth retardation. Early diagnosis remains challenging, as these symptoms often develop gradually. The disease also affects the carapace-protected skeleton and can result in pathological fractures in severe cases (5, 6, 9, 12).

Conventional radiography is a commonly used method for (subjectively) assessing bone density, but it is limited in its ability to detect early-stage bone mineral loss. Radiographic techniques often fail to detect significant bone changes until 40–50% of mineralization is lost (13). This is further complicated by the tortoise’s shell, which can obscure the underlying skeletal structures in a two-dimensional superimposition technique (12, 14). As a result, radiographs are of limited use for early disease detection, as well as for monitoring disease progression and treatment response (9, 12, 14). Nevertheless, in more advanced cases, radiographs can reveal characteristic patterns of bone pathology associated with MBD (5, 6, 11, 12, 15, 16). While these findings demonstrate the value of radiography in diagnosing and monitoring advanced MBD, they further emphasize the need for more precise, non-invasive imaging modalities to detect the disease at subclinical stages.

Advances in imaging techniques such as quantitative computed tomography (QCT) and dual energy X-ray absorptiometry (DEXA) have revolutionized our ability to noninvasively assess bone density in reptiles (14, 17–20). Although DEXA is considered the gold standard for bone mineral density (BMD) in humans, its use in chelonians and availability in practice are limited, with only two reports available on its application in this species (10, 21). QCT has previously been used for bone density evaluation in reptiles, including chelonians (15, 17, 18). It is regarded as more sensitive than conventional imaging modalities, offering additional insights into bone morphometry from which biomechanical parameters can be derived (14). CT generates high-resolution, three-dimensional images, enabling detailed examination of bone architecture and precise measurement of bone density with increased contrast resolution. Unlike plain radiography, CT eliminates superimposition of structures, a crucial advantage for chelonians due to their unique body structure and bony shell.

Moreover, QCT typically relies on external calibration using density phantoms to enable quantitative assessment of BMD (22). Phantomless calibration methods based on internal tissue references have been described in human clinical imaging (23, 24). Conventional QCT represents a dimensionless greyscale number, the Hounsfield Unit (HU), proportional to the degree of X-ray attenuation by the tissue.

Dual-energy CT (DECT) and spectral detector CT (SDCT) represent the cutting edge of bone density measurement. Detector-based SDCT utilizes a dual-layer detector system to differentiate between high- and low-energy photons originating from a single polyenergetic X-ray beam. The upper layer, composed of a yttrium-based garnet scintillator, absorbs low-energy photons, while high-energy photons reach the lower gadolinium-oxysulfide layer (25–30). This dual-energy acquisition enables simultaneous analysis of the photoelectric effect—dominant at photon energies below 100 keV—and the Compton effect, which prevails above 100 keV. By isolating these interactions, SDCT can calculate attenuation coefficients specific to different materials, facilitating spectral decomposition and the generation of various spectral image types (26, 29–31).

Unlike conventional CT, which may not differentiate materials with similar attenuation, SDCT can distinguish some materials based on their energy-dependent attenuation profiles (25, 28, 29). Dual-layer bone densitometry has shown promising results in both phantom studies and clinical human imaging (32–34). Importantly, SDCT offers a phantomless alternative to QCT, with the added advantage of retrospective spectral data reconstruction, eliminating the need for scan protocol preselection (25, 35).

The aforementioned spectral imaging technologies enable superior material decomposition and density analysis, leading to a more accurate understanding of bone mineralization. A comparative study assessing BMD measurements obtained from SDCT and phantom-based QCT has already been reported in dogs (36), demonstrating good agreement between both methods and supporting the accuracy and feasibility of spectral detector–based BMD quantification in veterinary patients.

Hermann’s tortoises (*Testudo hermanni*) are medium-sized, herbivorous reptiles, native to the Mediterranean and widely kept as pets in Europe (37). Like other reptiles, they exhibit a high susceptibility to MBD when maintained under suboptimal dietary or environmental conditions. This renders them a relevant species for investigating the relationship between husbandry practices and bone health.

Although the use of SDCT in tortoises is not yet widely reported, it shows promise for enhancing diagnostic capabilities beyond conventional QCT and DEXA.

This study investigates the feasibility of SDCT as a reliable method for BMD measurement in Hermann’s tortoises (*Testudo hermanni*). The overarching objective is to determine whether SDCT is suitable for early and reliable diagnosis of MBD and associated demineralization. Specifically, we aim to:

1. Quantify BMD differences between clinically healthy tortoises and those with varying severity of MBD, to assess the potential of SDCT for early-stage diagnosis.
2. Evaluate whether CT-derived linear morphometric shell ratios correlate with the severity of subjective shell softening.

We hypothesize that SDCT-derived ROI-based BMD values will distinguish healthy individuals from those with MBD. Additionally, we expect that increasing clinical MBD severity correlates with a decrease in global shell height-to-width ratios and an increase in the gular scute ratio, reflecting both overall flattening of the carapace and localized scute deformation.

## 2 Materials and methods

### 2.1 Study population

The study was carried out at the *Department of Small Animal Medicine at the University of Veterinary Medicine Hannover*. Hermann’s tortoises (*Testudo hermanni*) that met the inclusion criteria during the study period from April 2023 to September 2024 were enrolled. Inclusion criteria comprised the availability of both a physical examination and SDCT imaging. For each animal, collected data included diet and husbandry (housing, light regimen), age, body weight, sex, clinical signs, and available laboratory results at the time of presentation. Diet and husbandry conditions were assessed using a standardized anamnesis protocol based on established reptile medicine guidelines (6). Evaluation focused on risk factors for MBD, including inadequate dietary composition (e.g., inappropriate type and proportions of protein, vegetables, flowers or fruits which resemble low calcium or poor Ca: P ratio), insufficient UVB exposure, and suboptimal environmental temperature or humidity control. Based on clinical and diagnostic findings, tortoises were subsequently divided into two groups: a clinically healthy control group (group 0) and a group affected by MBD.

The control group was selected in accordance with the ethics commission’s approval, while the remaining tortoises, affected by MBD and undergoing CT examination due to shell softness, were enrolled in the study with the owners’ consent. The control group consisted of clinically healthy *Testudo hermanni* from an accredited wildlife rescue and rehabilitation center, maintained under standardized environmental conditions for at least 7 months. These individuals were considered clinically healthy based on normal physical examination findings, appropriate body condition, and unremarkable imaging results (whole body radiography, kidney ultrasonography), although minor deviations from reference intervals were observed in selected hematologic or biochemical parameters (Table 1).

**Table 1:** Available hematological and biochemical values for all animals.

Group	Animal	Hct %	ALT (U/L)	GLDH (U/L)	AST (U/L)	CHE (U/L)	Uric acid (mmol/L)	Ion Ca (mmol/L)	TCA (mmol/L)	P (mmol/L)
0	1	30	1	**3.2**	40	302	4.59	1.06	**2.03**	1.3
0	2	30	1	**2.6**	34	**721**	**7.22**	1.22	2.31	1.05
0	3	30	1	**2.6**	32	426	**6.3**	1.2	**2.12**	1.17
0	4	31	1	**2.5**	30	**930**	4.09	1.28	2.39	0.58
0	5	30	1	**3.2**	44	380	3.24	1.37	2.37	0.79
0	6	30	2	**6.3**	39	396	4.56	1.19	**2.12**	**1.27**
0	7	25	1	0.9	9	126	1.88	1.32	2.63	0.53
0	8	20	1	2	16	150	4.09	1.06	3.06	1.15
0	9	21	1	1.1	9	295	1.27	1.28	2.65	0.81
0	10	20	1	**9.9**	20	495	3.11	1.23	3.21	0.92
0	11	23	1	**4.4**	19	136	4.59	1.24	2.86	0.6
0	12	20	1	1.3	13	401	3.71	1.08	2.71	0.91
1	13	28	3	**12**	**68**	395	4.47	1.9	3.35	0.59
1	14	x	x	x	x	x	x	x	x	X
1	22	x	x	x	x	x	x	x	x	X
1	23	30	1	0.8	19	458	2.58	1.64	4.43	0.87
2	15	21	1	0.8	29	152	3.88	1.34	2.7	0.71
2	16	x	2	1.7	46	169	4.96	1.68	4.73	1.08
2	20	22	4	**5.1**	44	95	**7.32**	1.17	2.23	**0.48**
3	17	x	x	x	x	x	x	x	x	X
3	18	x	x	x	x	x	x	x	x	X
3	19	x	x	x	x	x	x	x	x	X
3	21	34	2	**3.3**	38	205	2.1	1.46	2.75	0.87

x = no available data. Data deviating from reference values are printed in bold.

All animals of the MBD group revealed varying degrees of shell softening upon examination. Based on subjective palpation of shell rigidity, these tortoises were further subdivided into three categories according to the severity of shell softening: mild (group 1), moderate (group 2), and severe (group 3). All tortoises were examined by a clinician specializing in herpetological medicine (J. H.) with over 5 years of experience in the field. Tortoises were excluded from the study if they showed signs of other systemic illnesses unrelated to MBD or if they had incomplete imaging data.

### 2.2 SDCT image acquisition

Images were acquired using a third-generation spectral detector computed tomography system (SDCT; Philips IQon Spectral CT, Philips Healthcare, Germany).

Transverse images were obtained with a slice thickness of 0.67 to 1.0 mm, using both soft tissue and bone kernel reconstruction algorithms. The helical pitch was 0.5, with a gantry rotation time of 1 s, a peak kilovoltage of 120 kVp, and automated amperage ranging from 295 to 400 mAS and a matrix size of 512 × 512 pixels. Conventional 120 kVp images (soft tissue and bone kernel) and Spectral-based imaging (SBI) data, including virtual monoenergetic (monoE) images (from 40 to 200 keV), were reconstructed from the raw data. Conventional reconstruction algorithms and monoE images at 50 and 200 keV were employed to generate a BMD map. For image analysis, conventional bone window reconstruction algorithms and BMD maps were utilized.

The tortoises were examined while awake and unsedated. During SDCT scans, they were positioned in sternal recumbency and immobilized by securing their carapace to a plastic box with tape to remain immobile. The animals were allowed to move their head and limbs during the procedure. Acquiring SDCT images took approximately 90 s per tortoise, including the initial radiograph for localizing the scan area and the main scan. After the scan, each animal was promptly returned to its enclosure within the clinic. One post-mortem scan was performed on a tortoise that had been euthanized before the CT examination due to its worsening condition. Euthanasia was carried out under deep sedation using intramuscular injection of ketamine (10 mg/kg) and medetomidine (0.15 mg/kg). After confirmation of loss of reflexes and nociception, the final intracardiac administration of pentobarbital (900 mg/kg) was performed.

### 2.3 Image analysis and bone density measurement

BMD was assessed using SDCT BMD maps derived from spectral imaging, which had to be generated prior to analysis. The BMD maps were calibrated using data from a previous study that used a calibration phantom (KP 70, Lot KP 03/3; Scanco Medical AG, Switzerland) (36, 38), as commercially available automated BMD mapping is currently unavailable. The phantom contains four cylindrical inserts with known hydroxyapatite (HA) concentrations (38), serving as a reference standard. The use of spectral data relies on calculating BMD by converting conventional HU through a linear model. For this purpose, the HU were measured in the actual HA concentration in milligrams per cubic centimeter (mg/cm^3^) in the four phantom tubes from conventional CT reconstructions.

For calibration, isotropic, circular, three-dimensional (3D) volumes of interest (VOIs) were drawn within four phantom inserts (100–800 mgHA/cm^3^) from a sample of 10 patient scans from a previous comparative study (36). The mean HU values from the 50 and 200 keV images were used to generate a regression model, which was applied pixel-wise to estimate the bone volume fraction. The resulting fractions were converted to BMD using the known densities of the phantom inserts, yielding a quantitative BMD map in which each pixel represents BMD in mg/cm^3^.

The technology behind BMD maps employs spectral material decomposition of monoE images to measure BMD in mg of HA per cubic centimeter (HA/cm^3^). MonoE images at 50 keV and 200 keV were generated from SBI datasets and served as the basis for this decomposition. These images simulate monochromatic X-ray images by separating the photoelectric and Compton scattering components from data captured by the dual-layer detector (39). The HU values at each energy level were derived through a weighted combination of these components.

Image analysis was performed using ITK-SNAP (version 3.6.0; Penn Image Computing and Science Laboratory, University of Pennsylvania),^1^ an open-source software tool for manual and semi-automatic segmentation of 3D medical images (40, 41). BMD maps and conventional images were loaded as transparent overlays in the software to facilitate macroscopic alignment of bone boundaries between conventional bone windows and BMD maps during ROI placement. Multiplanar reconstructions were applied for the quantitative evaluation.

BMD values were directly obtained from the spectral BMD maps in mgHA/cm^3^. The software provides automated measurements of mean values and standard deviations (SD), the total volume (mm^3^), and the total voxel count for each ROI. In addition to the mean values, the individual pixel values within the ROI were also determined to facilitate quantitative analysis using histograms.

ROI placement was performed by a single observer, a radiologist in training (A. H.), under the direct supervision of a board-certified veterinary radiologist (K. M.).

**Figure 1:**
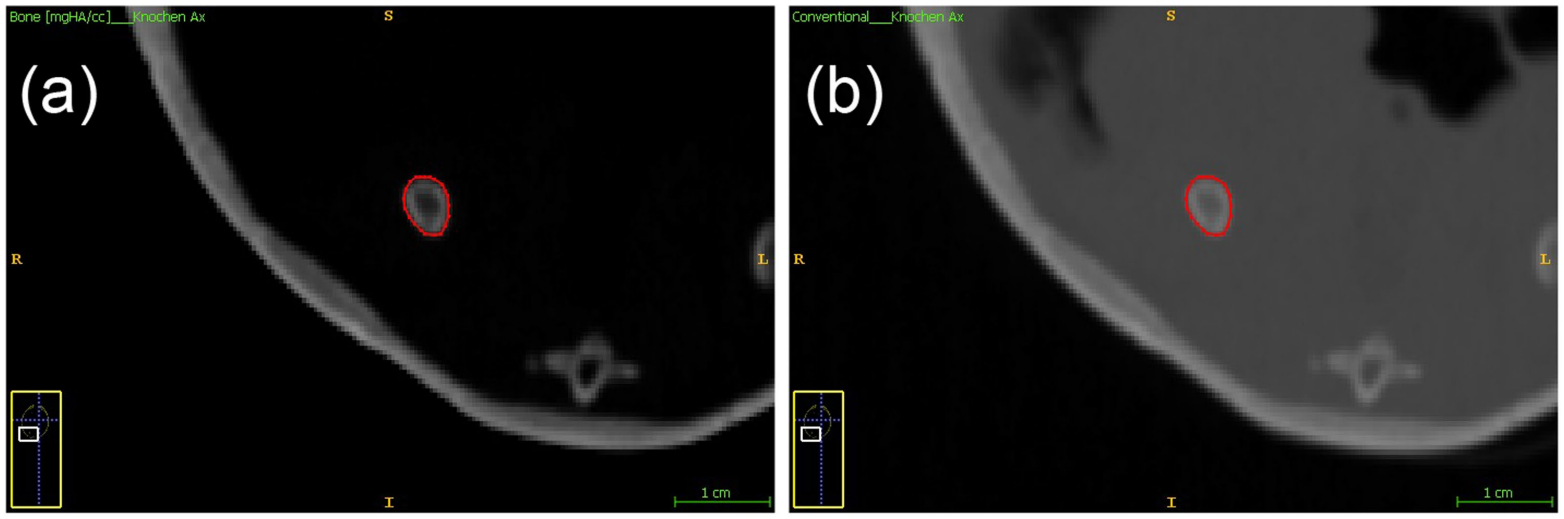
Modified ITK-SNAP images demonstrating ROI placement for BMD analysis: **(a)** SDCT BMD map of the mid aspect of the ilium in the dorsal plane, and **(b)** the corresponding conventional reconstruction a polygonal ROI (outlined in red) in the right ilium.

Five two-dimensional, smooth, curved, freehand polygonal ROIs were positioned at specific osseous locations within each tortoise. These included the mid-diaphyseal regions of the scapula and ilium on both sides, identified in the dorsal plane (Figure 1), and the mid-length of the gular scute of the plastron, examined in the transverse plane. To standardize the diaphyseal measurements, the total lengths of the scapula and ilium were measured, and the midpoints of each were used as the designated sites for ROI placement.

The ROIs covered the entire bony structure at each site, including both cortical and trabecular compartments, to determine total BMD. When possible, a statistical assessment was performed of both sides at the measurement locations on the scapula and ilium. No measurements were taken in areas where motion artifacts compromised the procedure. For each ROI, quantitative data were collected, including mean BMD, SD, voxel and pixel counts, pixel size, and individual pixel values within the ROI.

**Figure 2:**
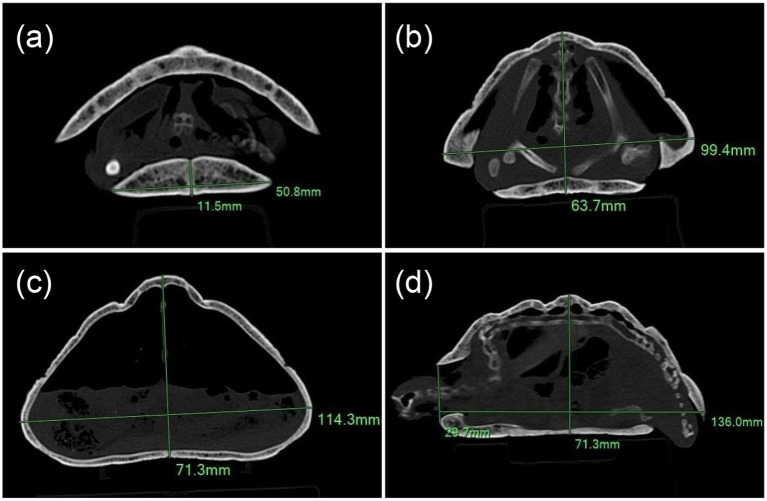
CT-based linear morphometric measurements performed on multiplanar reconstructions. Representative examples of image-derived linear measurements obtained from the CT datasets: **(a–c)** Transverse reconstructions showing **(a)** gular height and width; **(b)** shell height and width at the level of the shoulder girdle; **(c)** maximum body height and width; and **(d)** craniocaudal length measured in the sagittal plane.

Linear morphometric measurements were performed directly on the CT datasets. Measurements were obtained on multiplanar reconstructions using the software’s digital distance measurement tool. The maximum carapace length, width, and height of each tortoise were recorded. Additional measurements were taken at the level of the shoulder girdle and at the mid-point of the gular scute. At the shoulder girdle and the maximum shell height level, the maximum external shell dimensions (height and width) were measured, whereas at the gular site measurements were limited to the gular scute. Tortoise length was measured in the sagittal and dorsal planes, width in the dorsal and transverse planes, and height in the transverse and sagittal planes (Figure 2).

### 2.4 Statistical analysis

The data obtained by ITK-SNAP was directly collected in MS Excel and transferred to R using the readxl library for analysis. Statistical analyses were performed with the appropriate statistical software using R (version 4.2.2) and R Studio (2023.12.0, R Core Team, 2023, R Foundation for Statistical Computing).

Statistical analyses were conducted to compare differences between groups in BMD measurements and morphological ratios. Quantitative pixel data and mean BMD values were analyzed using the Wilcoxon rank-sum test (non-parametric), with Bonferroni correction applied for multiple comparisons.

Morphological ratios were similarly tested using the Wilcoxon rank-sum test with Bonferroni correction, following assessment of variance equality with Levene’s test.

A *p*-value ≤ 0.05 was regarded as statistically significant, with adjusted *p*-values shown after Bonferroni correction to account for multiple testing.

## 3 Results

### 3.1 Animal population and group classification

A total of 23 Hermann’s tortoises (*Testudo hermanni*) met the inclusion criteria and were enrolled in the study. The cohort comprised 12 clinically healthy animals (group 0) and 11 tortoises diagnosed with MBD of varying severity, divided as follows: group 1 (mild MBD): *n* = 4; group 2 (moderate MBD): *n* = 3; group 3 (severe MBD): *n* = 4.

Gender distribution was balanced in the healthy group (6 females, 6 males), whereas the diseased group comprised 4 females, 5 males, and 2 hatchlings of unknown sex.

The median age of the animals of group 0 was 15 years (estimated age ranged from 10 to 40 years), 10 (6–14 years) for group 1 and 10 (7–12 years) for group 2. Two individuals of group 3 were 11 months old, leading to a median age of 2.45 years (0.9–49 years). The median body mass was 1,138 g (range: 565–1,548 g) for group 0, 616 g (455–1,034 g) for group 1, 494 g (367–528 g) for group 2, and 178 g (34–1,061 g) for group 3.

For all animals, available hematologic, biochemical, clinical, radiographic and ultrasonographic findings are summarized in Tables 1, 2. The blood reference values used by our laboratory are derived from well-known sources in the literature (6, 42). Blood work was available for all 12/12 healthy animals and for 6/11 animals in the diseased group. Several diseased tortoises presented for follow-up examinations at our clinic, previous laboratory results were available and repeat sampling was not considered clinically warranted on the day of presentation. In some cases, the owners declined additional testing. In two hatchlings (animals 17 and 18), blood collection was not attempted due to their small body size and poor clinical condition, which precluded safe venous access. Ionized Ca concentrations were within the physiological reference range in all individuals. The lowest value (1.12 mmol/L) was recorded in a clinically healthy tortoise. The mean Ca: P ratio was 1.56 (range: 0.88–2.7) in the healthy group and 2.28 (range: 1.6–3.5) in the diseased group. Within the healthy group, several animals showed elevated P concentrations, and two of these (animals 2 and 3) also exhibited hyperuricemia, with uric acid concentrations of 7.22 mmol/L and 6.3 mmol/L, respectively. In the diseased group, hyperuricemia was observed in multiple individuals, including animal 16 (uric acid: 6.54 mmol/L) and animal 20, which had the highest uric acid concentration (7.32 mmol/L). Detailed hematological and biochemical values for all animals are presented in Table 1.

**Table 2:** Alterations in diet and husbandry.

Group	Animal	Diet	Husbandry	Pyramidial	Shell deformation	X-ray	Ultrasound of the kidneys
0	1	✓	✓	mild	X	✓	✓
0	2	✓	✓	mild	X	✓	✓
0	3	✓	✓	mild	X	✓	✓
0	4	✓	✓	X	X	✓	✓
0	5	✓	✓	moderate	X	✓	✓
0	6	✓	✓	mild	X	✓	✓
0	7	✓	✓	mild	X	✓	✓
0	8	✓	✓	moderate	X	✓	mild malperfusion
0	9	✓	✓	mild	X	✓	✓
0	10	✓	✓	moderate	X	✓	✓
0	11	✓	✓	mild	X	✓	✓
0	12	✓	✓	mild	X	✓	✓
1	13	✓	✓	mild	X	n.a.	n.a.
1	14	✓	✓	mild	X	n.a.	n.a.
1	22	✓	✓	moderate	enlarged	n.a.	n.a.
1	23	✓	X	mild	X	spongious	✓
2	15	✓	✓	moderate	X	n.a.	n.a.
2	16	✓	✓	mild	flat	✓	✓
2	20	X	X	moderate	X	moth eaten	mild malperfusion
3	17	X	X	X	flat	n.a.	n.a.
3	18	X	X	X	flat	n.a.	n.a.
3	19	X	X	mild	flat	n.a.	n.a.
3	21	✓	✓	mild	X	moth eaten	nephromegalie, malperfusion

Evaluated as sufficient = ✓, or insufficient = X, (presence of long-standing (>12 months) major dietary or husbandry deficiencies as defined in Methods) pyramidal growth evaluated as absent = X, mild, moderate, severe, shell deformation evaluated as absent = X, flat, or enlarged shell, absent radiographic and ultrasonographic studies (not available = n.a.), normal studies marked with a hook (✓), and abnormalities noted.

### 3.2 CT BMD data and ratios

A total of 110 anatomical sites used for BMD quantification were obtained from 23 chelonians, corresponding to the gular scute, scapulae, and ilia as defined for ROI-based BMD analysis. Five measurement sites were unavailable due to movement artifacts during scanning. The left scapula site was missing in animals 1, 2, 14, and 15, and the right scapula site was missing in animal 6. For bilaterally paired structures (Scapulae and Ilia), BMD values from left and right ROIs were compared to assess potential side-to-side differences. Significant side differences were observed in two animals (left and right scapula in animal 4 and left and right ilium in animal 19; *p* ≤ 0.0001). These values were excluded from further statistical analysis as they likely reflect measurement inconsistencies. No significant differences were observed in the remaining animals. Left and right measurements were therefore pooled for analysis. After these exclusions, 106 ROI-based measurement sites in 23 animals remained for analysis.

Animal 13 was identified as an upward value within group 1 but remained included according to the predefined clinical classification criteria (shell softness) and was therefore retained in all statistical analyses.

**Figure 3:**
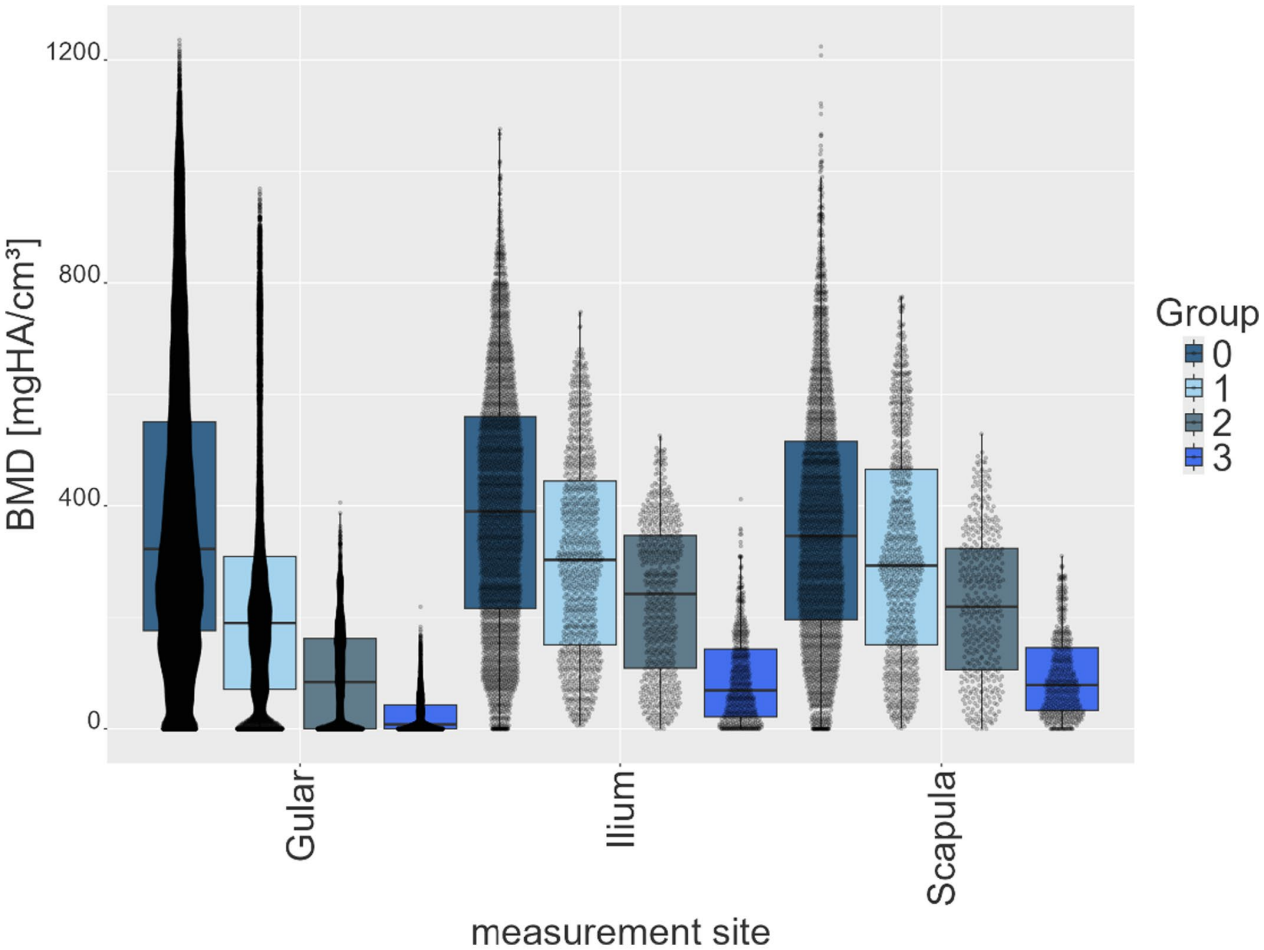
Box plots of all pixel data of each animal of each group (group 0–3) of shell softness for each measurement site (gular, ilium, scapula), expressed in bone mineral density (BMD) in mg HA/cm^3^.

**Figure 4:**
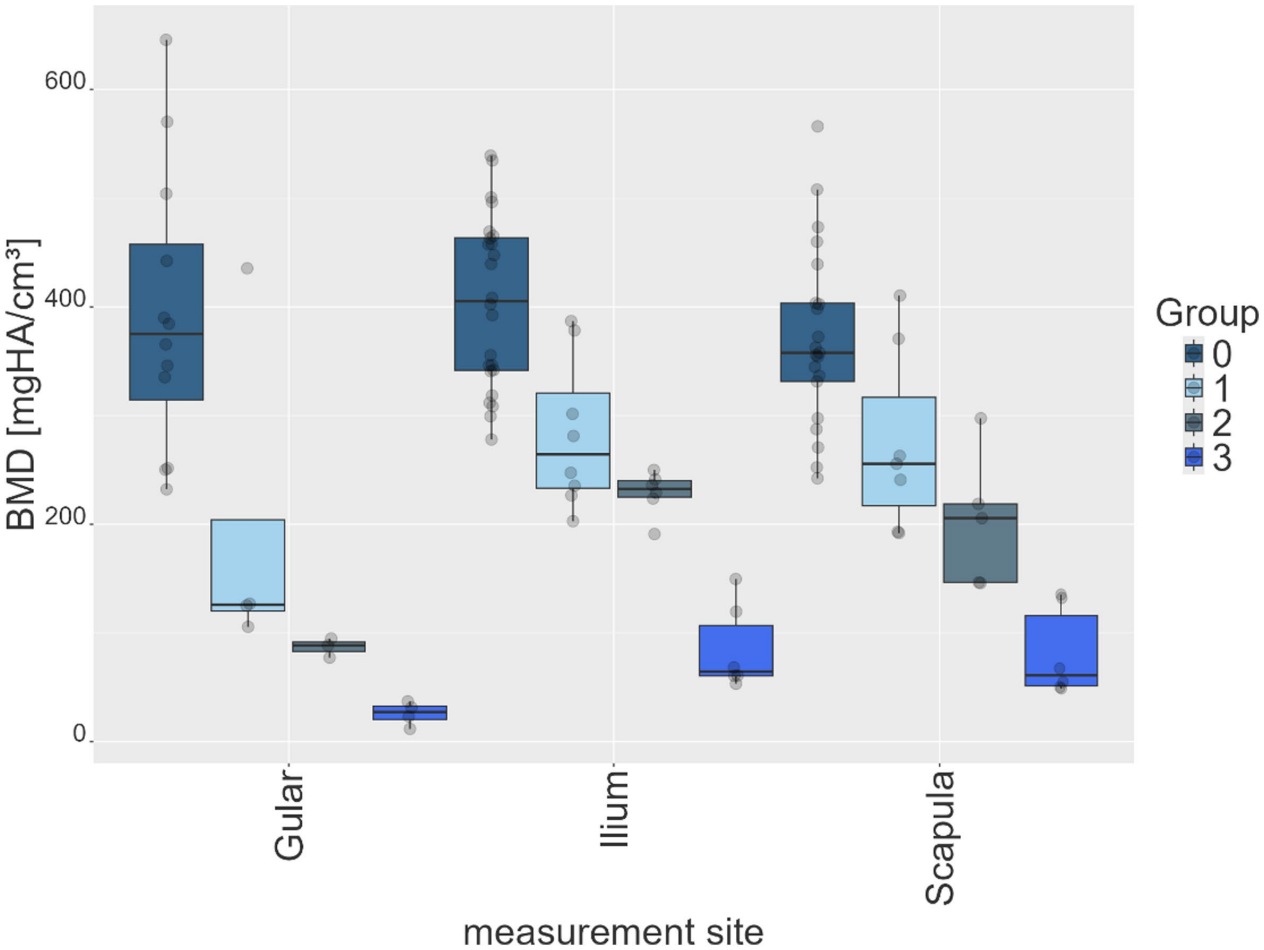
Box plots of mean values for each animal of each group of shell softness (group 0–3) at each measurement site (gular, ilium, scapula), expressed in bone mineral density (BMD) in mgHA/cm^3^.

Across all BMD measurement sites, a progressive decrease in recorded parameter values was observed with increasing degrees of shell softness from groups 0 to group 3 (Table 3; Figure 3). The mean values were highest in group 0 and lowest in group 3. At the gular site, mean values decreased from 382.4 mgHA/cm^3^ (group 0) to 24.7 mgHA/cm^3^ (group 3). The ilium and scapula sites showed a similar reduction, with mean values decreasing from 398.9 to 90.5 mgHA/cm^3^ and from 370.9 to 95.4 mgHA/cm^3^, respectively. Across all groups, the ilium and scapula regions consistently exhibited higher mean values than the gular region (Table 3; Figure 4). Median values closely aligned with these tendencies, with mean values exceeding medians in all observations. SD were highest in groups 0 and 1 (ranging from approximately 177 to 268) and markedly lower in the softer groups 2 and 3. Maximum and 99th-percentile values decreased steadily across groups, while the minimum and first-percentile values were close to zero at most sites, particularly in groups 2 and 3.

**Table 3:** Summary of important BMD-derived parameters (mgHA/cm^3^) per group and measurement site (gular, ilium, scapula) across all animals.

Group	Measurement site	Mean	Median	SD	Maximum	99th percentile	Minimum	1st percentile
0	Gular	382	323	268.2	1,236	1,062	0	0
0	Ilium	399	390	227.7	1,076	914.6	0	0
0	Scapula	371	346	225.7	1,224	967.1	0	0
1	Gular	237	190	223.2	969	877	0	0
1	Ilium	310	303	177.5	748	667.9	6	17.1
1	Scapula	315	293	195.3	775	749.9	1	16.5
2	Gular	92	84	86.8	406	278	0	0
2	Ilium	234	242	136.2	526	496.2	0	9.4
2	Scapula	223	219	131.3	529	480.5	0	6.8
3	Gular	25	8	32.1	219	137.4	0	0
3	Ilium	91	69	81.8	412	330.1	0	0
3	Scapula	95	79	74.7	310	274.9	0	0

**Figure 5:**
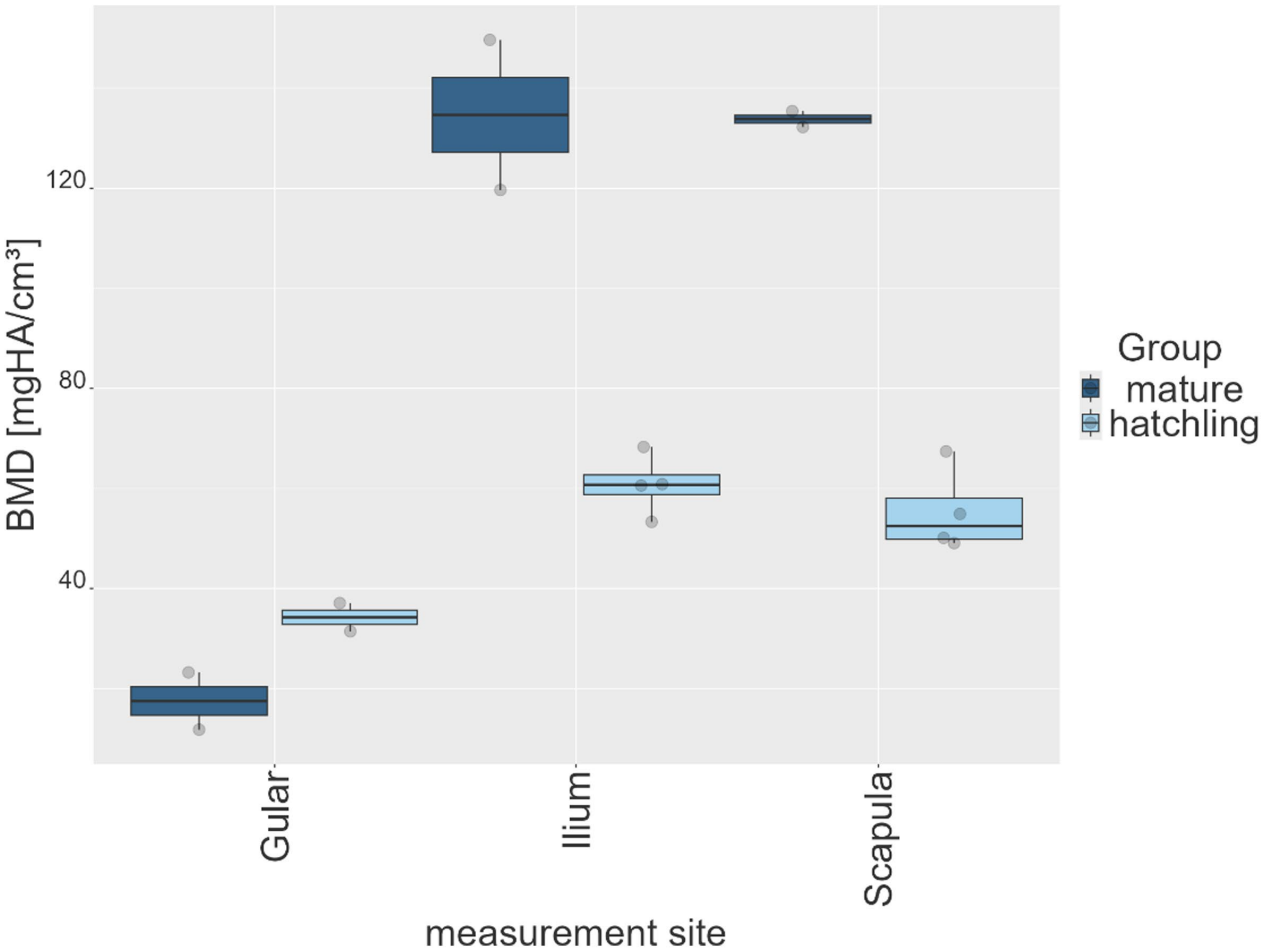
Sub-analysis of group 3 showing box plots of mean values for mature individuals (dark blue) and hatchlings (light blue) at each measurement site (gular, ilium, scapula), expressed in bone mineral density (BMD) in mgHA/cm^3^.

As group 3 included two hatchlings, an additional sub-analysis was performed to assess the potential impact of age on BMD. A comparison of the mean BMD values at the gular, scapular and iliac measurement sites was made using a box plot (Figure 5). The gular site showed comparable values, while juveniles demonstrated mildly higher gular values but noticeably lower values at the scapula and ilium compared with mature animals.

**Figure 6:**
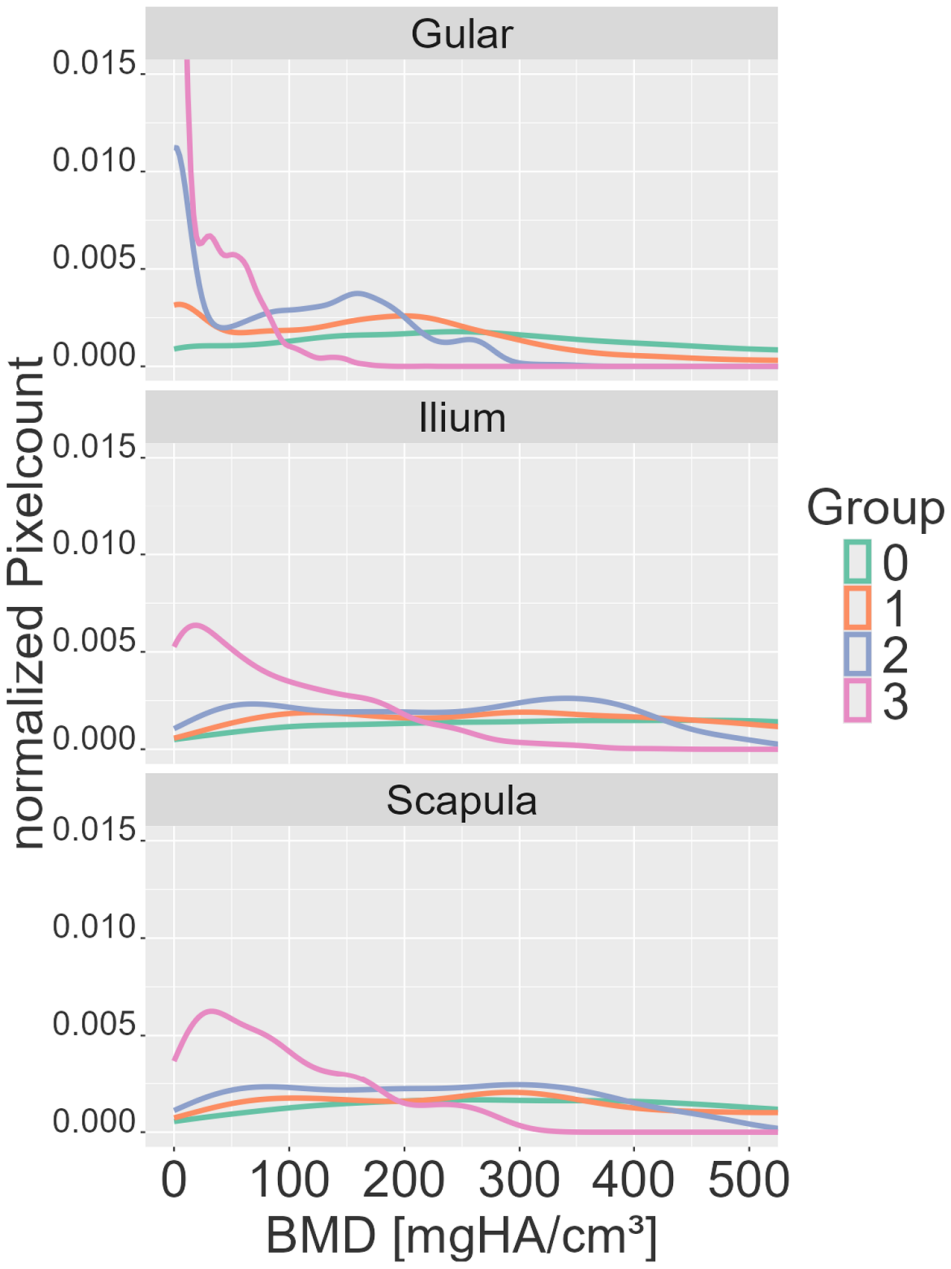
Histogram lines of pixel data for all animals in defined groups of shell softness (groups 0–3) at the different measurement sites (gular, ilium, scapula). The y-axis shows the normalized pixel count, and the *x*-axis shows the bone mineral density (BMD) of the measured SDCT data in mgHA/cm^3^.

Histograms of BMD quantification (Figure 6) showed that increasing shell softness was associated with an increase in low-value pixels and a reduction in high-value pixels. The healthy group (group 0) displayed the broadest and the most even distribution, with little to no skew in the data. The diseased groups (groups 1–3) showed progressively narrower distributions with an increasing degree of shell softness and increasing peaks at zero, especially in groups 2 and 3 at the ilium site, and in group 3 across all measurement sites. Histograms show right-skewed distributions with narrower ranges of BMD values, resulting in shorter tails from group 1 to group 3.

Mean values per animal per group across all measurement sites demonstrated significant differences between the healthy animals (group 0) and the diseased groups (groups 2–3). When specifically comparing healthy animals (group 0) with mildly affected animals (group 1), a significant difference was observed only at the ilium site, whereas differences at the scapular and gular sites were not statistically significant (Table 4). Differences at the gular site between diseased groups (groups 1–3) were non-significant. Comparisons between groups 1 and 2 at the ilium and scapular sites were also non-significant (Table 4).

**Table 4:** Results of the Wilcoxon test comparing mean values of all measurement locations (left and right combined) and degree of shell softness across all animals.

Measurement site	Group 1	Group 2	Adj. p-value	Level of significance
Gular	0	1	1.0	ns
Gular	0	2	7.2 × 10^−2^	*
Gular	0	3	1.8 × 10^−2^	**
Gular	1	2	1.0	ns
Gular	1	3	5.2 × 10^−1^	ns
Gular	2	3	1.0	ns
Ilium	0	1	1.4 × 10^−2^	**
Ilium	0	2	6.1 × 10^−5^	****
Ilium	0	3	6.1 × 10^−5^	****
Ilium	1	2	1.0	ns
Ilium	1	3	1.2 × 10^−2^	**
Ilium	2	3	3.6 × 10^−2^	**
Scapula	0	1	4.9 × 10^−1^	ns
Scapula	0	2	6.6 × 10^−3^	**
Scapula	0	3	1.2 × 10^−4^	****
Scapula	1	2	1.0	ns
Scapula	1	3	1.8 × 10^−2^	**
Scapula	2	3	7.2 × 10^−2^	*

Adjusted *p*-value after Bonferroni correction. Denotation of the level of significance: ns, non-significant; *p ≤ 0.05; **p ≤ 0.01; ***p ≤ 0.001; ****p ≤ 0.0001.

**Figure 7:**
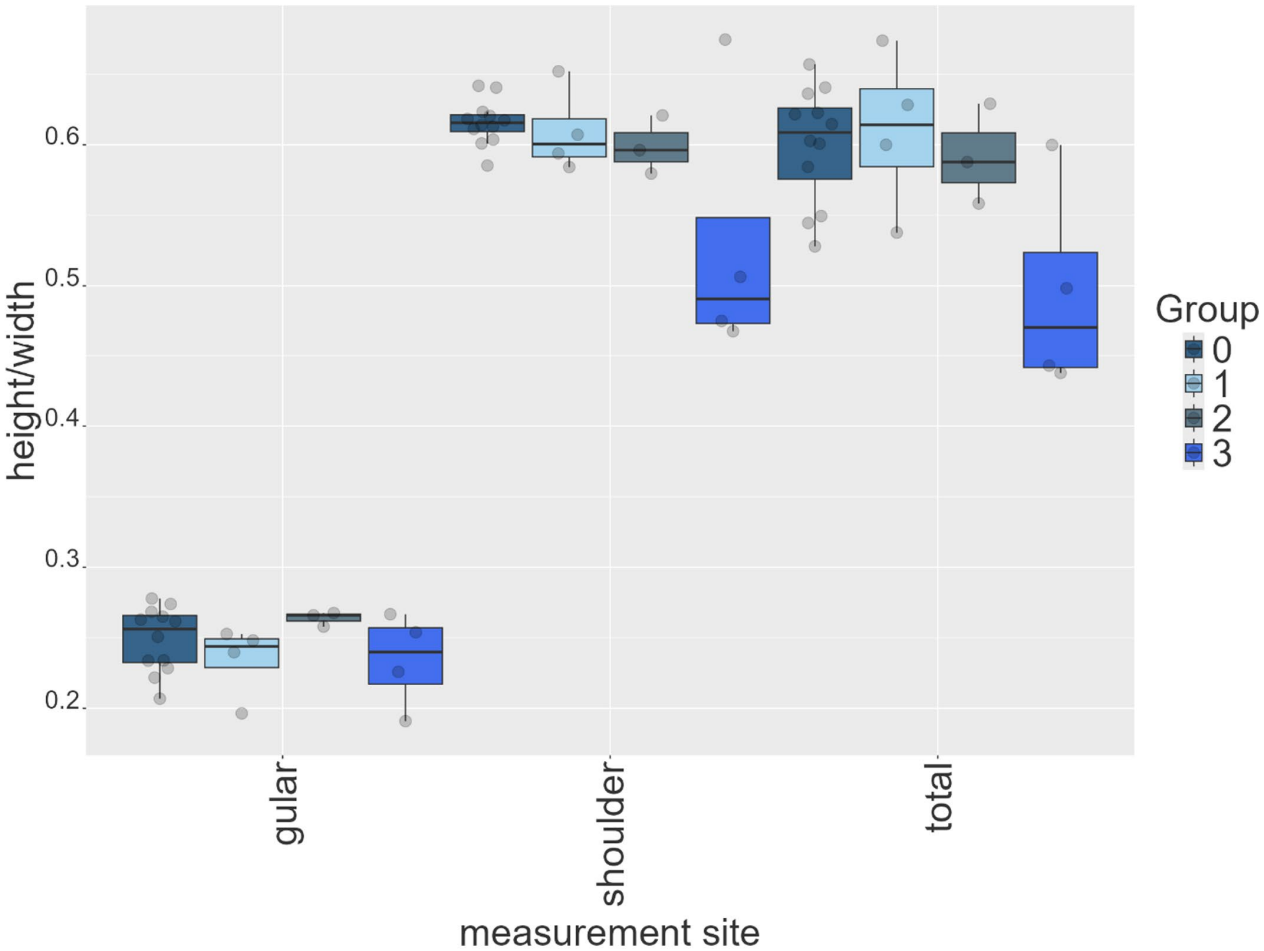
Box plots for measured height/width ratios at three different locations of all animals per group: from left to right: location at the gular scute, at the level of the shoulder girdle and the maximum diameter (total).

For morphometric linear distance measurements, Levene’s test indicated a significant difference in variance for the height-to-width ratio at the shoulder girdle (shoulder: *p* = 0.002), whereas the ratios measured at the maximum diameter (total: *p* = 0.483) and the ratio at the gular scute (gular: *p* = 0.105) did not show significant variance differences. Subsequent comparisons using the Wilcoxon test with Bonferroni correction revealed no significant differences in these ratios among the groups (*p* ≥ 0.05; Figure 7).

## 4 Discussion

This study demonstrates that SDCT can reliably quantify reductions in shell mineral density associated with increasing degrees of MBD in Hermann’s tortoises *(Testudo hermanni)*. These findings highlight the potential of SDCT as an objective diagnostic tool for early detection and quantitative assessment of demineralization, thereby extending beyond the limitations of subjective clinical and radiographic assessments of shell softness. This result is consistent with the conclusions of previous studies that employed advanced imaging techniques to evaluate demineralization and MBD in chelonians and other reptile species (15, 17, 18, 20, 21).

Across all anatomical sites, BMD values declined progressively from clinically healthy animals to those with advanced shell softness. This pattern reflects measurable changes in mineral composition and bone structure. The gular region showed the steepest decline at all three measurement sites, suggesting earlier or more pronounced involvement of this area. In contrast, the ilium and scapula retained comparatively higher values, potentially indicating delayed softening or structural differences compared with the gular region. This suggests that these regions may maintain rigidity or structural integrity for longer.

The reduction in standard deviations from group 0 to group 3 indicates that shells with advanced softening show lower variability within the ROI, consistent with a transition toward a more uniformly compliant structure (greater heterogeneity in harder shell categories and reduced variability at higher softness levels).

Histograms further demonstrated a right-skewed distribution, with a progressive shift toward low-value pixels in diseased animals and a narrowing of the distribution range, accompanied by a substantial accumulation of zero or near-zero values in moderate and severe cases. Together, these characteristics support a non-linear progression of shell demineralization, with the most pronounced changes occurring between the extremes of subjective grading (between groups 0 and 1 and between groups 2 and 3).

Quantitative BMD pixel values generally differentiated between clinically healthy and diseased individuals across all measurement sites. This confirms that radiological quantification can refine and objectify results from the clinical examination. However, some overlap was observed between adjacent clinical groups, particularly in animals with mild symptoms. This reflects the limitations of palpation-based classification and quantitative imaging in borderline cases. This was exemplified by one individual with BMD values closer to the healthy range, despite exhibiting clinical signs of shell softness (animal 13). Therefore, SDCT-derived BMD measurements are most valuable when used alongside clinical assessment, particularly when palpation alone is inconclusive, rather than as a standalone diagnostic criterion.

Mean BMD values differentiated healthy and diseased groups most significantly at the ilium, followed by the scapula and gular regions. Histogram analyses further emphasized the gular scute as a highly responsive site for distinguishing healthy from diseased animals, which may guide future diagnostic protocols. This apparent sensitivity may relate to regional anatomical differences of the shell. The gular scute represents a relatively thin and less robust portion of the plastron compared with the scapular and pelvic regions, which may render density changes more readily detectable at this site (19).

Morphometric analyses provided only limited insight into disease severity. A mild but consistent decrease in shoulder height–width ratios corresponded with increasing shell softness, supporting the hypothesis that progressive shell deformation accompanies MBD. However, the total shell height–width ratio showed inconsistent patterns. These inconsistencies may reflect the multifactorial influences on shell morphology. Low humidity significantly promotes pyramidal carapace growth, whereas dietary protein appears to have a lesser effect (43). Rapid growth induced by highly digestible diets has been shown to decrease bone mineralization, potentially contributing to abnormal shell morphology even without overt clinical MBD (44). Such environmental factors likely contributed to the variability observed in our study population. This variability points to the complex and multifactorial nature of shell morphology in tortoises (12, 43, 45, 46).

The relatively small sample size must be acknowledged to avoid overestimating the reliability and generalizability of the findings. Age of the animals was a heterogeneous parameter. The inclusion of two juveniles within group 3 further complicates interpretation, as age-related differences in BMD are well documented in previous studies (8, 47). In our sub-analysis, these juveniles demonstrated noticeably lower BMD values at the scapular and iliac sites compared with mature animals, suggesting that part of the observed reduction in group 3 may reflect physiological immaturity rather than disease severity alone. Absent age-stratified reference intervals, developmental variation may confound distinctions between physiological immaturity and pathological demineralization, and the relative contributions of age and disease cannot be clearly separated. Future studies should employ larger, age-balanced cohorts, ideally through multi-center studies, to establish reliable reference values for BMD in captive *Testudo hermanni* populations. Such normative data would serve as a critical benchmark for diagnosing and monitoring MBD.

Additionally, the classification of shell softness was based on subjective palpation by a single experienced examiner. Although this ensured internal consistency, the approach remains operator-dependent and does not account for potential inter-observer variability and may reduce reproducibility across clinicians. Subtle differences in shell rigidity on palpation, particularly in borderline cases between clinically healthy animals (group 0) and those with mild MBD (group 1), may have resulted in misclassification. Consequently, some mildly affected individuals may represent normal biological variation, whereas early or subclinical cases could have been included in the control group. This potential overlap is reflected in the presence of upward values and the limited statistical separation between adjacent severity groups (animal 13). Objective tools such as SDCT—and potentially mechanical shell hardness measurements, as previously described using a micrometer equipped with a tension-developing ratchet-release thimble (48)—could reduce such classification bias in future studies.

In addition to the classification-related restrictions, the study has several limitations. All tortoises in this study were captive and not kept under natural conditions. Consequently, our “healthy” reference group represents a health standard for *captive* animals rather than a wild-type baseline. This distinction is critical, as husbandry-dependent differences in growth and mineralization may influence both morphology and BMD. Furthermore, individuals with confirmed early-stage or subclinical MBD were not available. While SDCT clearly detected substantial reductions in BMD in moderate to severe disease, its sensitivity for identifying very early mineral loss remains uncertain. Mild cases may not yet show measurable BMD changes, or the current methodology may not detect such differences. Therefore, the ability of SDCT to reliably distinguish early-stage MBD from healthy individuals cannot be definitively established and should be addressed in future studies including early or preclinical cases and, ideally, free tortoises from their natural habitat in the wild.

Although bloodwork was available for part of the study population, values for ionized calcium, the Ca: P ratio, and uric acid did not improve diagnostic classification. This aligns with the existing literature, which reports that blood parameters often remain within normal ranges even in chelonians with confirmed MBD (6, 43, 49, 50). The limited diagnostic value of routine biochemical testing underscores the need for objective imaging-based methods. The absence of further microbiological or virological investigations precludes the possibility of an infectious etiology such as infectious lethal serositis (11, 51). Infections with picornaviruses, especially in young chelonians and hatchlings, have been associated with nephropathy and osteodystrophy. These conditions can lead to shell softening that clinically resembles MBD (52, 53).

A pathological examination could also not be performed due to the clinical nature of the study and the primary objective of ensuring the individuals’ survival. Nevertheless, these limitations do not affect the methodology used to quantify bone density as a causative factor in bone softening.

Finally, the current lack of vendor-provided software for automated spectral BMD quantification currently limits SDCT to research use. In this study, BMD maps required manual calibration and time-consuming ROI analysis using third-party software, which is not feasible in routine clinical workflows. Integrated, automated calibration, standardized BMD map reconstruction and analysis tools directly on the scanner workstation would be required for broader clinical implementation. To our knowledge, only one study to date has evaluated the SDCT-BMD-map method in veterinary medicine, comparing it with phantom-calibrated QCT (36). Once suitable clinical software becomes available, the diagnostic utility of SDCT for evaluating tortoises with suspected MBD is likely to expand significantly.

In conclusion, SDCT offers a promising, non-invasive and objective approach to quantifying shell and bone mineralization and assessing disease severity in chelonians. It enables differentiation between healthy and diseased individuals and may serve as a valuable tool for monitoring therapeutic outcomes. Larger, age-stratified cohorts, standardized grading protocols, and the establishment of captive reference values are essential to enhance diagnostic precision and support reptile medicine.

## Data Availability

The raw data supporting the conclusions of this article will be made available by the authors, without undue reservation.

## Footnotes

1
www.itksnap.org
